# LifePlanning: A Better Retirement Planning Option for Our Times

**DOI:** 10.1093/geroni/igaa057.368

**Published:** 2020-12-16

**Authors:** Rajiv Nagaich, Carol Redfield, Ben Harvill

**Affiliations:** 1 Aging Options, Federal Way, Washington, United States; 2 Aging Options, Seattle, Washington, United States

## Abstract

Ten thousand turn 65 daily. Majority look forward to retiring in the beginning and then become afraid of outcomes they often hear about- dealing with institutional care, becoming a burden, or running out of money. This is not because retirees do not plan, but despite of having planned their entire life for retirement. Many employers provide financial retirement planning such as a 401K plan. Individuals have relied on employee benefit plans to ready themselves, yet few are “very confident” about it. Two-thirds of retirees say their most recent employers did “nothing” to help them transition into retirement; 16% are “not sure” what their employers did. Many may be overlooking important factors in their strategies. Among retirees who currently have a retirement strategy, 85% have factored Social Security and Medicare benefits into their strategy. Most have included on-going living expenses (79%), total savings and income needs (57%) into their plan. Fewer than half have considered other critical factors (e.g., investment returns, ongoing healthcare costs, inflation, long-term care needs, tax planning, etc.). Only 9% have contingency plans for retiring sooner than expected and/or savings shortfalls. The truth is that education offered by employers tends to be traditional planning advice, which may not be enough to address the concerns retirees will have in retirement. To this, we introduce a multi-disciplinary LifePlanning Framework which takes a wholistic, integrated approach in addressing the many complex issues of retirement found in health, housing, finance, legal, and family. Our results may impact future practice, research, and policy.

